# Interference Mitigation for Visible Light Communications in Underground Mines Using Angle Diversity Receivers

**DOI:** 10.3390/s20020367

**Published:** 2020-01-09

**Authors:** Pablo Palacios Játiva, Milton Román Cañizares, Cesar A. Azurdia-Meza, David Zabala-Blanco, Ali Dehghan Firoozabadi, Fabian Seguel, Samuel Montejo-Sánchez, Ismael Soto

**Affiliations:** 1 Department of Electrical Engineering, Universidad de Chile, Santiago 8370451, Chile; pablo.palacios@ug.uchile.cl; 2 Department of Telecommunications Engineerings, Universidad de las Américas, Quito 170503, Ecuador; 3 Department of Computing and Industries, Universidad Católica del Maule, Talca 3466706, Chile; davidzabalablanco@hotmail.com; 4 Department of Electricity, Universidad Tecnológica Metropolitana, Av. Jose Pedro Alessandri 1242, Santiago 7800002, Chile; adehghanfirouzabadi@utem.cl; 5 Department of Electrical Engineering, Universidad de Santiago de Chile, Santiago 9170124, Chile; fabian.seguelg@usach.cl (F.S.); ismael.soto@usach.cl (I.S.); 6 CRAN, CNRS UMR 7039, Université de Lorraine, BP 70239 Nancy, France; 7 Programa Institucional de Fomento a la I+D+i, Universidad Tecnológica Metropolitana, Santiago 8330378, Chile; smontejo@utem.cl

**Keywords:** angle diversity receiver, inter-cell interference, signal combining scheme, underground mining, visible light communication

## Abstract

This paper proposes two solutions based on angle diversity receivers (ADRs) to mitigate inter-cell interference (ICI) in underground mining visible light communication (VLC) systems, one of them is a novel approach. A realistic VLC system based on two underground mining scenarios, termed as mining roadway and mine working face, is developed and modeled. A channel model based on the direct component in line-of-sight (LoS) and reflections of non-line-of-sight (NLoS) links is considered, as well as thermal and shot noises. The design and mathematical models of a pyramid distribution and a new hemi-dodecahedral distribution are addressed in detail. The performances of these approaches, accompanied by signal combining schemes, are evaluated with the baseline of a single photo-diode in reception. Results show that the minimum lighting standards established in both scenarios are met. As expected, the root-mean-square delay spread decreases as the distance between the transmitters and receivers increases. Furthermore, the hemi-dodecahedron ADR in conjunction with the maximum ratio combining (MRC) scheme, presents the best performance in the evaluated VLC system, with a maximum user data rate of 250 Mbps in mining roadway and 120 Mbps in mine working face, received energy per bit/noise power of 32 dB and 23 dB, respectively, when the bit error rate corresponds to $10^{-4}$, and finally, values of 120 dB in mining roadway and 118 dB in mine working face for signal-to-interference-plus-noise ratio are observed in a cumulative distribution function.

## 1. Introduction

The underground mining industry is considered a priority industry for many governments worldwide, so it has made great technological advances in its infrastructure and work environment [1]. However, there are problems related to the mining environment that make work difficult and endanger the lives of mining workers, such as the presence of poisonous substances, toxic gases, and corrosive water, as well as dust. Another latent problem in underground mines occurs when there are landslides and it is necessary to know the location of workers who need to be rescued [2]. A way to mitigate these problems is by designing specific communications systems for underground mining environments. Therefore, among the characteristics that these communication systems must have are: transmission of reliable and real-time information on the location and tracking of workers when a disaster occurs, transmission of smoke and hazardous gas detection data, information monitoring of mining machinery, and so on [3,4]. These details added to the hostile environment of a mining company, make the design of underground mine communications systems a challenging task, which, if properly implemented, would guarantee work safety and optimize the productivity of the mines.

Based on the mining environment’s features, the transmission priorities and the behavior of the transmission method, there are several categories studied in underground mining communications, among which are wired communication systems (coaxial, fiber-optic, and twisted pair), radio communication systems, carrier current communication systems and the combination of some of these systems (hybrid systems) [5,6]. It is understood that the underground mining environment is hostile; thus, wired communication systems are susceptible to damage and cannot be a reliable mode of communication in underground mines. Furthermore, radio communication systems and carrier current communication systems also cannot be used in underground mines due to attenuation of the signal. Even though, the underground mining environment needs good lighting that meets international standards, this problem remains without efficient solutions. All these challenges are presented as opportunities for proposals for emerging and complementary communications systems that optimize communication and lighting of underground mining environments. To fulfill these requirements, the concept of visible light communication (VLC) has been introduced for underground mining communication [7,8,9].

There are several benefits of using VLC technology compared to other wired and wireless communication techniques: unlicensed spectrum in which visible light operates; reasonable prices of the components that make up the VLC system; high data transfer rates; and, the most important VLC characteristic, the resistance to electromagnetic interference, providing reliable communication in addition to reducing the risk of accidents due to poor ambient lighting [10,11]. Another important characteristic of VLC systems is the security that it presents in the data transmission, specifically in the physical layer [12,13]. As is well known, several current research outputs presents VLC system models for indoor environments, which, due to their characteristics [14], are perfectly applicable to mining environments. The main components of an underground mining VLC system are, first; light-emitting diodes (LEDs), which are cold light sources; as well as having a long service life, they are used to form LED lamps that could be installed on the top surface of underground mines; and second, photo-diodes (PDs) that could be installed on the worker’s helmet, to create a VLC link between workers and mining infrastructure, or between workers, depending on the required application. Channel modeling is also an important stage for efficient, reliable, and robust VLC system design. There is increasing research associated with characterization of indoor VLC channels that can more accurately model a VLC link, in addition to considering all the factors that can influence the link behavior [11,14,15].

Compared to typical VLC indoor systems, VLC environments for underground mines are more complex to model. A typical underground mining scenario consists of small tunnels with irregular walls, so the VLC channel can be modeled considering the line-of-sight (LoS) and non-line-of-sight (NLoS) components, provided by the direct signal emitted by the LED (optical transmitter) to the PD (optical receiver) and the signals reflected on the walls that also reach the receiver, respectively [9,16]. Further, due to the light characteristics, as well as the LEDs and PDs, intensity modulation (IM) techniques in the transmitter and direct detection (DD) in the receiver must be implemented in these systems. According to all these features, a complete optical propagation model of the signal received at the receiver must also be proposed. To date, there has been little research regarding underground mining VLC models that involve these details [17].

Among the problems that may occur in underground mining VLC systems, it is well known by many studies that, when the optical receivers are in the overlapping area of two adjacent optical cells, the signal-to-interference-plus-noise-ratio (SINR) of these receptors could decrease considerably, due to the existence of strong interference produced by the inter-cell interference (ICI) phenomenon, which severely degrades the system performance [18,19]. Also, specifically in underground mining environments, there may be fluctuations in SINR, which are caused by the NLoS components due to reflections of light on irregular surfaces, which also affects the reduction of system efficiency. A considerable amount of scientific literature has been published that, presents solutions to efficiently mitigate the ICI [20,21]. Recently, a new technique, based on angle diversity receiver (ADR), has been introduced in typical indoor VLC environments [18,19,22,23,24,25]. An ADR consists of multiple narrow-field-of-view (FoV) PDs, which, when combined, result in a large-overall-FoV and coverage as a single PD. Each of the narrow-FoV PDs can be selected or combined through signal combining schemes, thereby reducing the interference effect of the signals that produce ICI. Nevertheless, to the best of the authors’ knowledge, there is no previous work addressing ICI mitigation problems and SINR fluctuation reductions in the field of underground mining VLC, which implies that solutions to these problems like those of ADRs, have not been tested in mining scenarios either.

In the following subsections we present several models related to the underground mining VLC channel. Then, state-of-the art solutions for the ICI problem in VLC, specifically involving ADR schemes, are presented. Finally, we briefly present works related to the direct application of the ADR solution in hostile VLC environments.

### 1.1. VLC Channel Models Applied to Underground Mining Environments

Three proposals for the characterization of the underground mining VLC channels are presented in [9,16,26]. In these proposals, a general VLC channel model for underground mining environments is presented, which involves the application of two communication scenarios, called mining-to-miner (M2M) and infrastructure-to-miner (I2M). Regarding the mathematical model of the VLC channel, the LoS and NLoS components are considered in their respective impulse responses. The analysis carried out in these works is based on the determination of the channel path loss, the large-scale fading, and scattering. The lighting parameter of mining environments is also briefly reviewed. The results determine an empirical path loss model of the VLC channel. For their contributions, these works are considered the starting point for our research. However, they still present opportunities for improvement. Among the details that must be addressed is the inclusion of complete thermal noise and shot noise models in the optical propagation model, the analysis of the interference that these scenarios may suffer, due to the number of LEDs that may exist, the SINR analysis of the environment and solutions for its improvement, among others.

An interesting analysis of VLC in hostile environments is applied in [8], where an energy coupling model of a VLC system applied to a coal mine is addressed, specifically a theoretical study of the effects of coal dust particles on VLC and the problem of optical signal degradation is performed. As a result, an optimal position of the optical transmitter is found, to maximize the coupled energy. Note that when addressing a specific problem of mining VLC environments, as only addressing the problem from the optical transmitter, it leaves open the possibilities for other solutions in optical reception.

An application of a light fidelity (LiFi) communication system for underground mines is presented in [27], which allows the sending of emergency messages in the mine. Another application of a VLC-based alarm system for mining environments is presented in [28]. Finally, several works that applied localization in mining environments based on VLC systems are presented in [29,30,31,32]. As we can see, the largest number of works related to VLC in underground mines, are security applications, location, or brief proposals of channel models that can be improved; thus, this presents an opportunity to analyze other problems present in these scenarios, such as the decrease of the ICI or the improvement of system performance.

### 1.2. Angle Diversity Receivers in VLC Systems

ICI is a major problem that seriously degrades the overall performance of indoor VLC systems [33,34]. Due to this issue, the SINR of the optical receivers would be considerably low, which is mainly caused by the existence of strong interference. To address this problem, the ADR based solutions are introduced for the first time to improve the efficiency of indoor MIMO-VLC systems in [24]. The ADRs can achieve a high-rank channel matrix by decorrelating the optical channel efficiently. The PDs are used with different FoVs to reduce the channel correlation in VLC systems. Furthermore, the concept of ADRs is also used to reduce the ICI and SINR fluctuations in indoor multi-cell systems, as evidenced by relevant studies [18,19,22,25,35,36,37]. In these investigations, the benefits of using ADRs in VLC systems as well as the general design of ADRs are analyzed to optimize the communication capacity. Among the general schemes of ADRs analyzed are those of pyramidal and hemispherical geometry along with their mathematical models, optimized location of the PDs, use of signal selection and combination schemes, and general tests in indoor VLC systems [38,39,40,41]. Despite growing research on ADRs, the existing benchmark schemes do not optimize the structure of ADRs, and its performance is not fully explored. Other works focus on increasing the capacity and spectral efficiency of the system area; however, an analysis of the maximum data rate that these ADRs can reach has not been performed. Finally, the most important aspect that is relevant to our work is that these schemes have not yet been tested in underground mining VLC environments; therefore, to the best knowledge of the authors, this is the first time that solutions of this type are implemented in VLC mining environments.

### 1.3. Application of ADRs in Hostile VLC Environments

After an in-depth literature review, no direct applications of ADRs in underground mining VLC environments were found. However, a recent work involving angular diversity selection schemes in hostile environments, specifically tunnel construction, is presented in [42]. This paper presents a characterization of the optical channel for tunnels, implementing PDs arrayed with angular diversity to improve the symbol error rate (SER) in reception. Although the tunnel construction environment is similar to the underground mine, the dimensions of the scenarios and the location of the transmission LEDs are different. Besides, the performance analysis in this work is preliminary, without giving greater depth to solutions of angular distributions of PDs or evaluation of other VLC system parameters.

In this work, we expand the work in [43], extending its application in a VLC underground mining scenario, and considering two typical mining environments: mining roadway and mine working face [9,16]. Moreover, a new ADR geometric distribution is proposed, called hemi-dodecahedron ADR, to evaluate its performance to mitigate the ICI and compare it with the pyramidal ADR presented in [22]. Three signal combination schemes are included with the ADRs [44]: select best combining (SBC), equal gain combining (EGC), and maximum ratio combining (MRC). To the best of our knowledge, this is the first time that the problem of ICI in VLC underground mining environments has been analyzed, and that ADRs are presented as a solution to mitigate the ICI in these VLC mining scenarios. The contributions of this work are the following.

1. A detailed underground VLC system model, in which, further to considering LoS and NLoS components with their channel impulse responses and channel direct current (DC) gains, thermal noise, and shot noise are also considered. These parameters affect underground mining environments to a greater extent. Furthermore, a brief analysis of the lighting in the proposed mining scenarios is done.
2. An analytical framework of a pyramidal ADR is performed, based on a regular pyramidal polyhedron, where the PDs are located on the lateral triangular faces, pointing in different directions, which mitigates the ICI and minimizes fluctuations of the SINR.
3. A new ADR structure is proposed, it is based on a regular hemi-dodecahedron, which consists of a PD located on its upper pentagonal face and five PDs located on its lateral pentagonal faces. This design acquires the best characteristics of the pyramidal and hemispherical ADRs existing in the state of the art, with the objective of reducing the ICI.
4. The impact of applying the proposed ADRs, in the improvement of the overall system performance, is measured by using the following metrics: the user data rate (UDR), the SINR, shown as a cumulative distribution function (CDF), and the BER, together with the SBC, EGC, and MRC schemes, validating them with Monte-Carlo simulation results. Although few authors have addressed the issue of VLC in underground mines, some of these metrics have not been evaluated in previous studies, these being necessary to verify the ICI mitigation.

The remainder of this paper is organized as follows. In Section 2, the VLC system model for underground mining is presented in detail. In Section 3, we contextualize the ICI in VLC underground mining environments and describe the analytical frameworks of the ADR solutions presented for this work. In Section 4, the signal combining schemes to be implemented with the ADRs are mathematically described. Section 5 evaluates and compares the solutions presented in the underground VLC mining system, based on several established metrics. Finally, relevant conclusions are presented in Section 6.

## 2. VLC System Model for Underground Mining

**Figure 1:**
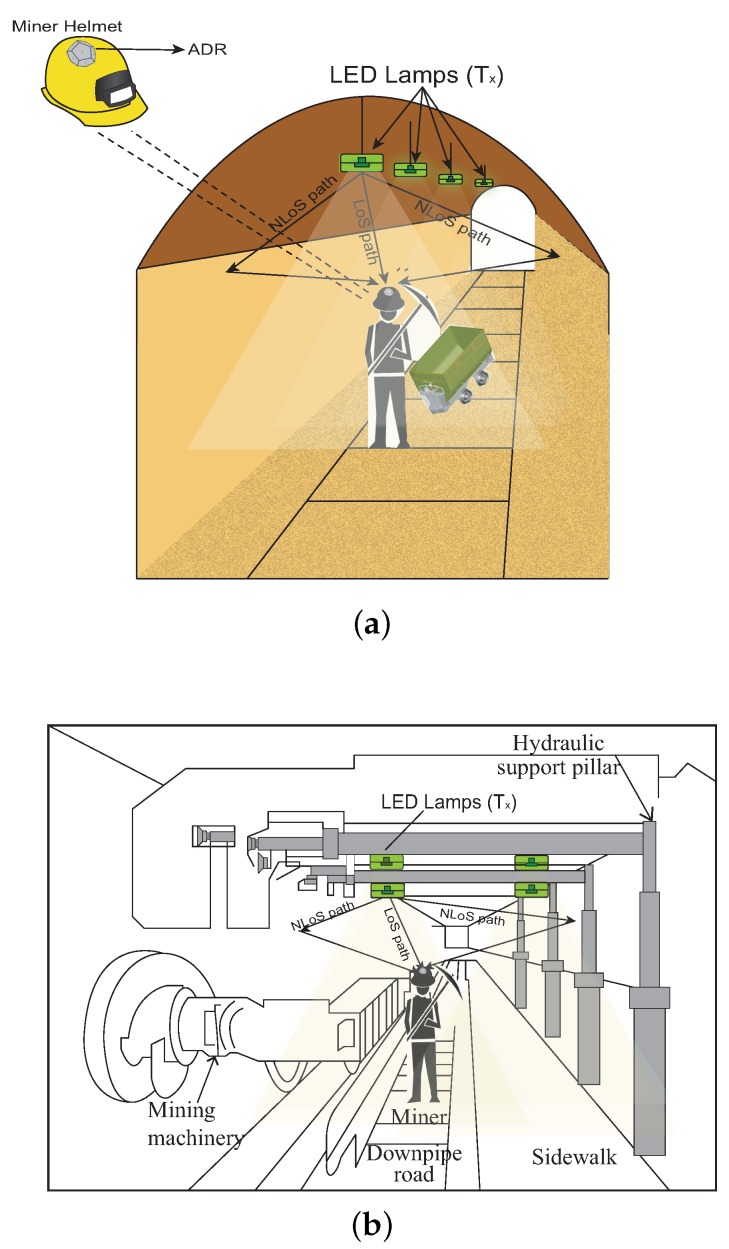
Underground mining VLC System Model. (**a**) Scheme of the mining roadway environment with the LED lamps row location and (**b**) scheme of the mine working face with the quadrature location of the LED lamps.

In general, underground mines are divided into two environments: one where mining workers can walk and carry the extracted materials, called “mining roadway” [9,16], and another where mineral extraction work is carried out, called “mine working face” [9,16]. The model of a mining system is presented in Figure 1, which we describe below.

1. Mining Roadway [9,16]: This part of the mining environment is generally a tunnel or a narrow chamber, where workers are mobilized and materials extracted from the mine are transported. In addition, it is characterized by being very narrow, with a short visibility distance and irregularities in the shapes of the walls and roof. In our work, for simplicity purposes, we will use a horseshoe-shaped environment to represent this mine section, whereby the LEDs will be located in a row on the roof, equidistant from each other, to provide communication and lighting simultaneously, see Figure 1a. Notice that since this region does not allow work to take place, environmental interference caused by external factors will not be preponderant.
2. Mine Working Face [9,16]: In this mine section, material extraction works are carried out by using specialized equipment. In the extraction process, electromagnetic radiation, water vapor, and flammable gases are produced. For these phenomena, wireless RF communication is not the best option, since it can produce interference or be absorbed. We will assume that this environment is rectangular, whereby the LEDs are located in rectangular form, equidistant from the center of the work section, to provide lighting and communication, see Figure 1b.

**Figure 2:**
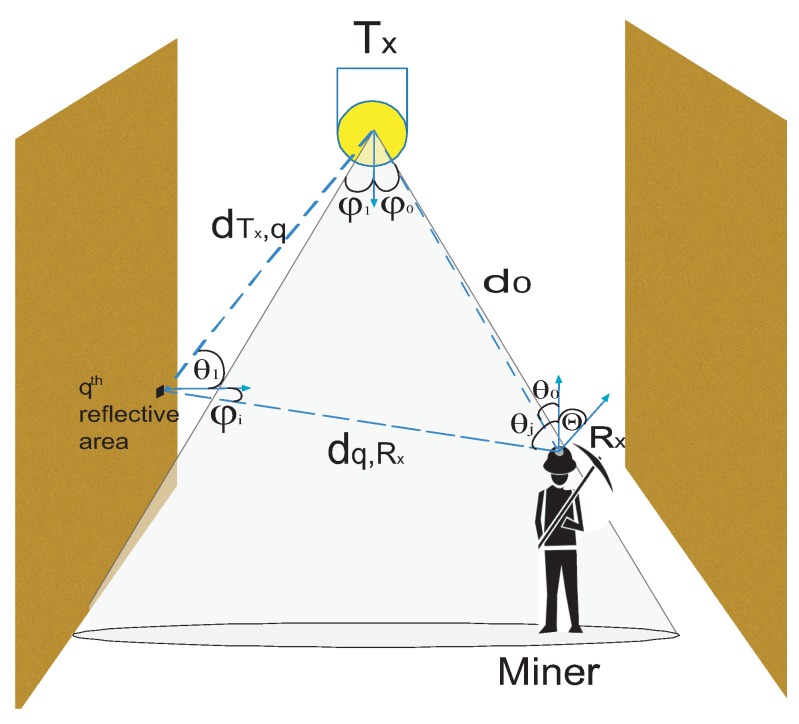
Geometry of the LoS and NLos propagation model.

Both sections are equipped with LED lamps, and in order to optimize the effect of communication, the light intensity in the spaces should be distributed as uniformly as possible. Therefore, multiple LED lamps must be installed on the roof of each mining section. The dimensions, LED lamp locations, and key parameters of the proposed underground mining VLC system are detailed in Table 1, located in Section 5. Taking as reference the communication scenarios proposed in [16], for our study, we will adopt the I2M communication scenario. This is because the LED lamps will be fixed on the ceiling of the mining sections; thus, acting as optical base stations that provide communication and lighting. In addition, this communication model considers LoS and NLoS propagation components, which favors us to propose a complete channel mode, as we can see in Figure 2.

### 2.1. Optical Transmitter

To optimize the effect of communication in underground mines, the light intensity in the space should be distributed as uniformly as possible. According to the work in [9], multiple optical transmitters (LED lamps) should be installed of the ceiling of underground mines. In this manuscript, we consider a specific area portion of the mining scenarios presented, with $T_{x}$s located in different positions depending on the mining scenario analyzed. When comparing the distance between the LED’s array, the size of the LED lamps can be ignored. Therefore, the LED’s array can be considered as a single entity and the luminous power of each LED array is equal to the sum of the LED lamps’ luminous power. In our work, and based on [9], each LED array consists of 10×10 identical LED lamps. In addition, to simplify the calculation, LED lamps in the same LED array transmit the same signal. Thus, we generalize the concept that each LED’s array is considered as an optical transmitter $T_{x}$. Assuming that each LED lamp of the $T_{x}$ has the same generalized Lambertian radiation pattern, and as this model is widely used for the light emission distribution of the LEDs [11], the angular distribution S(ϕ) of the intensity radiation pattern is given as [11]

(1) $$S\left(\phi \right)=\left\{\begin{array}{cc} \frac{m+1}{2\pi }\cos^{m}\left(\phi \right) & \mathrm{if}\;\phi \in [-\frac{\pi }{2},\frac{\pi }{2}]\\ 0 & otherwise \end{array}\right.,$$

where ϕ is the incidence angle and *m* is the Lambertian mode number, which is related to the half angle $\phi_{1/2}$ of the half power emitted by the $T_{x}$; *m* acquire the form of [11]

(2) $$m=\frac{-ln(2)}{ln\left[cos(\phi_{1/2})\right]}.$$

The maximum intensity corresponds to $\phi_{1/2}$=0. When *m* is increased, the $T_{x}$ beam may be more directional.

### 2.2. Channel Gain for the Underground Mine VLC Environment

Given the characteristics involved in a mining environment, the location of the $T_{x}$ on the roof of the mining scenarios, we will call the optical receivers $R_{x}$. These will be located at the top of the mining workers’ helmets, which agrees with the scenario of the proposed VLC model I2M. Due to the mobility that workers have in mining environments, the optical link will be different according to the location at some time of the $R_{x}$. Consequently, several gain components for the optical channel should be considered. In our manuscript, the LoS path is adopted as the first and principal gain component, as it is the largest source of energy obtained from the $T_{x}$. Short-distance LoS links are often modeled as linear attenuation and delay [11]. Also, these are considered non-frequency selective and the path loss depends on the square of the distance between the $T_{x}$ and the $R_{x}$ [11]. Therefore, the impulse response of the LoS component applied to underground mining environments can be written as [11]

(3) $$h_{0}\left(t\right)=\frac{A_{eff}\left(m+1\right)}{2\pi d_{0}^{2}}\cos^{m}\left(\phi_{0}\right)\cos\left(\theta_{0}\right)\delta \left(t-\frac{d_{0}}{c}\right),$$

where $A_{eff}$ is the effective signal collection area, define as $A_{eff}=A_{p}T_{s}\left(\theta_{0}\right)\;g\left(\theta_{0}\right)$. Here, $A_{p}$ is the physical area of the $R_{x}$, $T_{s}\left(\theta_{0}\right)$ is the optical filter gain, $g(\theta_{0})$ is the optical concentrator gain, exposed in Section 2.4, $d_{0}$ is the distance between $T_{x}$ and $R_{x}$, $\phi_{0}$ is the LoS transmission angle, $\theta_{0}$ is the reception angle, *c* is the speed of the light in free space, and $\delta (t-\frac{d_{0}}{c})$ is the Dirac’s function, which represents the signal propagation delay. Here, it is assumed that $\phi_{0}$ < $90^{\circ }$ and $\theta_{0}$ < Θ, where Θ is the $R_{x}$ FoV. We can also define the channel DC gain of the LoS path, according to the following expression [11]:

(4) $$H_{0}=\int_{-\infty }^{\infty }h_{0}\left(t\right)dt=\frac{A_{eff}\left(m+1\right)}{2\pi d_{0}^{2}}\cos^{m}\left(\phi_{0}\right)\cos\left(\theta_{0}\right)rect\left(\frac{\theta_{0}}{2\Theta }\right),$$

where rect(·) knows as the rectangular function, represents the signal reception decision function.

Not only does the LoS path contribute energy in mining scenarios, since in these hostile environments multiple factors affect the energy received in the $R_{x}$, such as non-uniform walls, dust particles or extracted materials, reflectivity of the ceiling, walls or others objects, dimensions of the environment, position and orientation of the $T_{x}$ and $R_{x}$, among others; these factors also create NLoS and diffuse links. Therefore, the optical path loss is more difficult to predict for NLoS links.

To model the gains from the reflections that may exist in an underground mining environment, non-smooth surfaces can be modeled by dividing their surface area into a number of small surface elements, where each area reflects a fraction of incident light energy. Further, each small surface element can be taken as a differential reflective element, which, through its integration, will give us the total reflective area. In general, the fraction of energy is proportional to the reflection coefficient of the surface material.

The light behavior in an underground mining environment can be modeled in two parts. The first is the path of light between a $T_{x}$ and a $q^{th}$ reflective area, whose impulse response is given as [9,11,16].

(5) $$h_{1,q}\left(t\right)=\int \frac{A_{ref}\left(m+1\right)}{2\pi d_{T_{x},q}^{2}}\cos^{m}\left(\phi_{1}\right)T_{s}\left(\theta_{1}\right)g\left(\theta_{1}\right)\cos\left(\theta_{1}\right)rect\left(\frac{\theta_{1}}{\Theta }\right)\delta \left(t-\frac{d_{T_{x},q}}{c}\right)dA_{ref},$$

where $A_{ref}$ is the reflective surface area, $d_{T_{x},q}$ is the distance between $T_{x}$, and the $q^{th}$ reflective area. In the second, the $q^{th}$ reflective area behaves like a light source, which is reflected on to the $R_{x}$. Besides, since there are many areas that can cause reflections in a mining environment, it is necessary to generalize them. Therefore, the impulse response of this path can be written as [9,11,16]

(6) $$h_{i}\left(t\right)=\sum_{q=1}^{Q}\frac{A_{eff}\;\rho_{q}\;\left(n+1\right)}{2\pi d_{q,R_{x}}^{2}}\cos^{n}\left(\phi_{i}\right)\cos\left(\theta_{j}\right)rect\left(\frac{\theta_{j}}{\Theta }\right)\delta \left(t-\frac{d_{q,R_{x}}}{c}\right)h_{i-1,q}\left(t\right),$$

where *i* represents the total number of light reflections, $d_{q,R_{x}}$ is the distance between the $q^{th}$ reflective surface and $R_{x}$, *Q* is the total number of reflective areas, $\rho_{q}$ is the reflection coefficient of the $q^{th}$ reflective area, and *n* is the Lambertian order of the reflective area. The *n* value is a function of the radiation angle $\theta_{ref}$, which represents half the intensity of the $q^{th}$ reflective element expressed as $n=[log_{2}{\left(cos\left(\theta_{ref}\right)\right]}^{-1}$. For our study, we assume $\theta_{ref}$=$60^{\circ }$ [22,23]. By using the expression to obtain the channel DC gain of the LoS path, the expression for the channel DC gain of the NLoS path acquires the form of [9,11,16]

(7) $$H_{i}=\int_{-\infty }^{\infty }h_{i}\left(t\right)dt=\sum_{q=1}^{Q}\frac{A_{eff}\;\rho_{q}\;\left(n+1\right)}{2\pi d_{q,R_{x}}^{2}}\cos^{n}\left(\phi_{i}\right)\cos\left(\theta_{j}\right)rect\left(\frac{\theta_{j}}{\Theta }\right)H_{i-1,q}.$$

Finally, the overall DC gain for underground mining VLC systems is the sum of the LoS and NLoS components, namely, [22,23]

(8) $$H=H_{0}+\sum_{i=1}^{I}H_{i},$$

where *I* is the number of reflections considered in the NLoS path. The graphic detail of the LoS and NLoS paths described in this section can be seen in Figure 2.

### 2.3. Optical Propagation Model

Considering that among the characteristics of the mining environments, there must be multiple $T_{x}$s and $R_{x}$s, there will also be multiple PDs distributed in $R_{x}$. Thus, we will denote $N_{T_{x}}$ as the total number of $T_{x}$, whose indexes for any $T_{x}$ is $a=1,2\cdots ,N_{T_{x}}$. We also define the number of PDs in the $R_{x}$ as $N_{PD}$, whose indexes for any PD is $i=1,2\cdots ,N_{PD}$. Therefore, the equivalent base-band model that we will use to describe an underground mining IM/DD VLC link is given as follows [23]

(9) $$y_{p}\left(t\right)=\tau \sigma \xi P_{a_{op}}x_{a_{op}}\left(t\right)⊗h_{op,p}\left(t\right)+\sum_{a=1,a\neq a_{op}}^{N_{T_{x}}}\tau \sigma \xi P_{a}x_{a}\left(t\right)⊗h_{a,p}\left(t\right)+n_{R_{x}}\left(t\right),$$

where $y_{p}\left(t\right)$ is the electrical signal received by the $p^{th}$ PD, τ is the electric-optical conversion efficiency σ is the PD responsivity, ξ is the $T_{x}$ modulation index, and ⊗ is the convolution operator. As there are multiple $T_{x}$ and $R_{x}$ with several PDs, we will assume for this model that only one $T_{x}$ should be selected as the optimum, where $a_{op}$ is the index of this $T_{x}$. In later sections, we will explain in more detail the selection or combination mechanisms of several signals emitted by the $T_{x}$ of the system. Therefore, $x_{a_{op}}\left(t\right)$ and $x_{a}\left(t\right)$ are the electrical signals transmitted from $T_{x_{op}}$ and $T_{x_{a}}$, respectively, $h_{op,p}\left(t\right)$ is the channel impulse response between the $Tx_{op}$ and the $p^{th}$ PD, $h_{a,p}\left(t\right)$ is the channel impulse response between the $T_{x_{a}}$ and the $p^{th}$ PD, $P_{a_{op}}$ and $P_{a}$ are, respectively, the average output optical power from $T_{x_{op}}$ and $T_{x_{a}}$.

An important point considered in our work for a complete propagation model is the noise. We will consider $n_{R_{x}}$ as the additive noise in $R_{x}$, which includes two types of noise that particularly affect mining environments, shot noise $\sigma_{R_{x},shot}^{2}$ and thermal noise $\sigma_{R_{x},thermal}^{2}$ [45,46]. Both noises can be modeled as additive white Gaussian noises (AWGNs), whose variances are determined by the following expressions [11,45,47],

(10) $$\sigma_{R_{x},shot}^{2}=2q\gamma P_{R_{x}}B_{n}+2qI_{bg}I_{2}B_{n},$$

(11) $$\sigma_{R_{x},thermal}^{2}=\frac{8\pi \kappa T_{k}}{G}\eta A_{p}I_{2}B_{n}^{2}+\frac{16\pi^{2}\kappa T_{k}\Gamma }{g_{m}}\eta^{2}A_{p}^{2}I_{3}B_{n}^{3}.$$

$\sigma_{R_{x},shot}^{2}$ contains two components; the former corresponds to photon fluctuation noise or quantum noise, where *q* is the electronic charge, $B_{n}$ is the noise bandwidth, and $P_{R_{x}}$ is the total power received by $R_{x}$ that contains the power of the desired $T_{x}$ and the power by the inter-symbol interference (ISI) caused by the others $T_{x}$. Therefore, $P_{R_{x}}$ can be represented as $P_{R_{x}}$= $P_{a_{op}}$+$\sum_{a=1,a\neq a_{o}p}^{N_{T_{x}}}$$P_{a}$. The latter corresponds to the dark current and excess noise, where $I_{bg}$ is the background current due to the ambient light and the noise bandwidth factor is $I_{2}$, equal to 0.562 [11,45,47]. In the thermal noise $\sigma_{R_{x},thermal}^{2}$ the former and latter components represent feedback-resistor noise and FET channel noise, respectively. Here, κ is Boltzmann’s constant, $T_{k}$ is absolute temperature, *G* is the open-loop voltage gain, η is the fixed capacitance of PD per unit area, Γ is the Field-Effect Transistor (FET) channel noise factor, $g_{m}$ is the FET trans-conductance, and the noise bandwidth factor is $I_{3}$, equal to 0.0868 [11,45,47].

It is worth noting that the shot noise is proportional to the power that $R_{x}$ receives, and thermal noise is depends on $A_{p}$ in a sophisticated way, so these factors are important when implementing optical components in underground mining environments.

### 2.4. Optical Receiver

**Figure 3:**
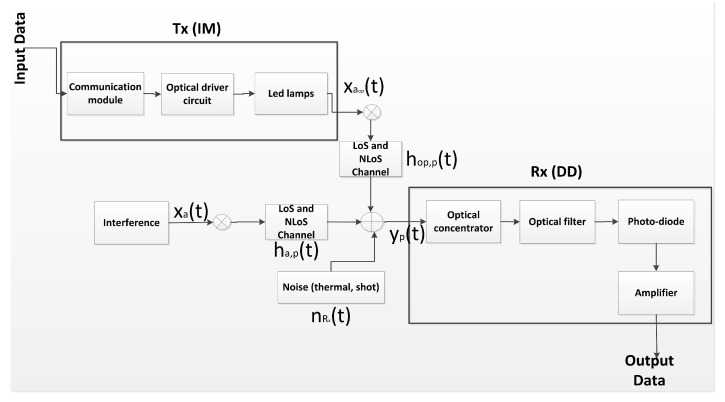
Block diagram of the underground mining VLC system.

VLC systems typically use PDs in the reception stage, which is composed of a non-imaging concentrator (lens) and a physical area $A_{p}$. PDs collect the incident power produced by the light intensity of $T_{x}$. The optical gain of a PD, $g(\theta_{0})$, based on a non-imaging concentrator, is given as follows [11]

(12) $$g\left(\theta_{0}\right)=\left\{\begin{array}{cc} \frac{\eta^{2}}{sin^{2}\left(\theta_{0}\right)}, & \mathrm{if}\;0\leq \theta_{0}\leq \Theta \\ 0, & \mathrm{if}\;\theta_{0}\geq \Theta \end{array}\right.,$$

where η is the refractive index of the concentrator. Along with the PD lens, an optical transmission gain band-pass filter, $T_{s}\left(\theta_{0}\right)$, is also added. The block diagram of the complete VLC system is shown in Figure 3.

As mentioned, one of the goals of this research is to study the problem of ICI in underground mining VLC environments. As such, we will focus on the proposal of three types of $R_{x}$ as possible solutions to ICI. The first proposal is a single-PD in the $R_{x}$, which is pointing up. In the second solution, a pyramidal ADR is presented that consists of four PDs located on the side faces of the pyramid. Finally, the third solution analyzes a new ADR hemi-dodecahedron scheme that consists of one PD located at the upper base pointing up and five PDs located on the side faces of the polyhedron. Note that all $R_{x}$ are located at the top of the mining workers helmets. A rigorous mathematical analysis of the ICI problem and the proposed solutions is presented in the following section.

## 3. ICI Problem in Underground Mining VLC Environments and ADR-Based Solutions

To contextualize the ICI problem in underground mining VLC environments, a brief analysis of this phenomenon is described below. Then, the theoretical analysis of the solutions proposed in this research, based on ADRs, is presented.

### 3.1. Contextualization of the ICI Problem in Underground Mining VLC Environments

**Figure 4:**
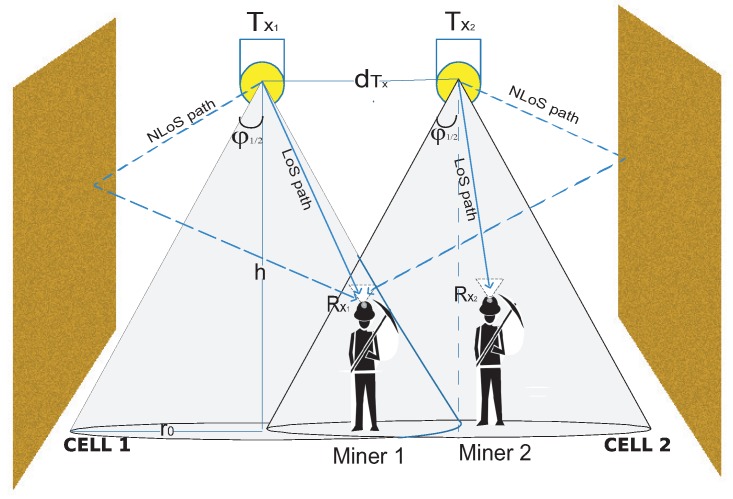
ICI presented in a multi-cell VLC system.

In a noisy VLC scheme such as a mining environment, there must be multiple $T_{x}$, because it is necessary to have full lighting and communication coverage in the scenarios studied. Figure 4 presents an example where two adjacent optical cells are shown, in which there is an overlapping area in their respective coverage areas. Therefore, the ICI in these schemes is always present. To present this problem, we must identify the most important parameters involved in the existence of ICI. According to Figure 4, we will consider two $T_{x}$ that maintain a distance $d_{T_{x}}$ from each other. In addition, it is considered $\phi_{1/2}$ and the height between the roof where the $T_{x}$ are located and the mining floor, called *h*.

The optical cells produced by $T_{x_{1}}$ and $T_{x_{2}}$, assuming that all the $T_{x}$ of the mining scenario have the same physical parameters, cover a circular area of radius $r_{0}$. By trigonometric relationship, $r_{0}$ acquires the form of

(13) $$r_{0}=h\;tan\left(\phi_{1/2}\right).$$

Therefore, in order for the coverage areas of the optical cells to overlap and produce ICI, it must be met that $d_{T_{x}}$ < 2$r_{0}$, in other words, the distance between the $T_{x}$s is less than the diameter of the optical cell.

In general, in a mining VLC scenario, there are several $T_{x}$s that produce optical cells that overlap each other; as such, the ICI is a challenge that must be addressed to improve the efficiency of the underground VLC mining system. To contribute to the mitigation of this impairment, the use of multiple PDs in $R_{x}$ is proposed to reduce the ICI, which are located in geometric distributions, termed as ADR. Benefits of applying these structures in $R_{x}$ can be summarized as follows.

1. The geometric structure of the ADR means that the PDs located there point in different directions and at different angles; this diversifies the reception of the optical signals.
2. ADRs provide a fixed coverage area that is based on the FoV of each PD. This becomes narrower when the number of PDs is increased. This benefit allows reduction of the LoS ICI caused by neighboring $T_{x}$s.
3. Being light reflections of a dispersive nature, having a narrower FoV, the ICI NLoS can be reduced, because most of the light components received by reflections would be rejected.
4. By increasing the number of PDs in $R_{x}$, according to their location in the ADRs, greater granularity can be obtained, which improves the ability to suppress the ICI. However, among the factors that should be considered when choosing the geometric distribution and the number of PDs to use, must be included the complexity of the ADR design, the cost and processing time of the received signals, and the physical manufacturing cost of these models.

Based on these benefits and features, we present below the design and analysis of two ADR schemes, one of pyramid structure, and a new scheme based on a hemi-dodecahedral distribution.

### 3.2. ADR with the Pyramid Structure

A pyramidal ADR distribution was chosen based on its simplicity as well as design feasibility, complying with the principle that the FoV of the PDs must point to different directions for a greater area of total coverage at reception. Besides, the overlap between the FoVs of the PDs is minimized and its structure is compact, thus making the separation between PDs small.

**Figure 5:**
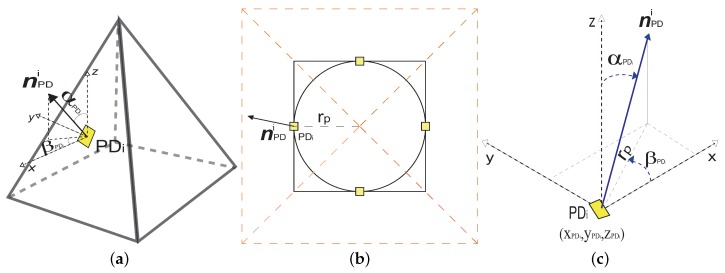
General structure of the pyramidal ADR. (**a**) Side view, (**b**) top view, and (**c**) coordinate system of the PDs.

This distribution is known as pyramidal because it consists of four PDs characterized by having the same FoV [22,43]. These PDs are located in the center of the triangular side faces of the pyramid, leaving the base free for its placement in the helmet of the miners. Also, the PDs point in the direction of the normal vectors of the pyramid lateral surfaces, as we can observe in Figure 5a. Furthermore, it is also possible to observe in Figure 5b that the PDs are uniformly organized in a circle of radius $r_{p}$, which is located in the horizontal plane that intersects in the center of the pyramidal distribution.

For a better understanding of the orientation and location of the PDs in the ADR, based on normal PD positioning vectors $n_{PD}$. We will define a hybrid coordinate system, (x,y,z,α,β), where (x,y,z) are the Cartesian coordinates of the position of the normal vector; α is the angular coordinate of position with respect to the *z*-axis, which we will call the elevation angle and will be delimited in α∈[0,π]; and β is the angular coordinate of position with respect to the *x*-axis, which we will call the azimuth angle and will be delimited in β∈[0,2π] [22,43]. Therefore, the $n_{PD}^{i}$ of a $PD_{i}$ located at $\left(x_{PD_{i}},y_{PD_{i}},z_{PD_{i}}\right)$, will be given by [22,43]

(14) $$n_{PD}^{i}=\left(x_{PD_{i}},y_{PD_{i}},z_{PD_{i}},\alpha_{PD_{i}},\beta_{PD_{i}}\right)\;\;\;\;\;\;\;\;for\;\;1\leq i\leq 4.$$

Details of the coordinate system and its position are depicted in Figure 5c. In order to define the orientation of the PDs in the ADR, following the pyramidal geometry, it is possible to deduce that all the PDs will have the same elevation angle, which is also the same elevation angle of each of the pyramid side faces. As it is a regular polyhedron, we can mention that ∀i∈[1,4]: $\alpha_{PD_{i}}$=$\alpha_{PR}$, where $\alpha_{PR}$ is the elevation angle of the side faces of the pyramid. As well as for pyramid symmetry, the normal vectors of the pyramid’s lateral faces will be equidistant from the center of the radius circumference $r_{p}$; therefore, $\beta_{PD_{i}}$ for each $PD_{i}$ will be evenly distributed, according to the following expression [2,18],

(15) $$\beta_{PD_{i}}=\frac{2\pi (i-1)}{4}.$$

Therefore, the PDs will be specifically oriented in 0, π/2, π, and 3π/2. By considering the center of the pyramid distribution as a reference point, it is possible to define the Cartesian coordinates for the $PD_{i}$, as follows [2,18]

(16) $$\left(x_{PD_{i}},y_{PD_{i}},z_{PD_{i}}\right)=\left(x_{PD}+r_{p}cos\left(\beta_{PD_{i}}\right),y_{PD}+r_{p}sen\left(\beta_{PD_{i}}\right),z_{PD}\right),$$

where $\left(x_{PD},y_{PD}\right)$ are the coordinates in (x,y) measured from the center of the pyramid, and $z_{PD}$ is the ADR height. Using as reference the hybrid coordinate system illustrated in Figure 5c, it is then possible to obtain the relation between $r_{p}$ and $\alpha_{PD_{i}}$, given by $r_{p}=sin\left(\alpha_{PD_{i}}\right)$. The relation between $z_{PD}$ and $\alpha_{PD_{i}}$ given by $z_{PD}=cos\left(\alpha_{PD_{i}}\right)$. Thus, it is possible to leave expressed the Cartesian coordinates for the $PD_{i}$, in terms of its orientation angles, as follows

(17) $$\left(x_{PD_{i}},y_{PD_{i}},z_{PD_{i}}\right)=\left(x_{PD}+sin\left(\alpha_{PD_{i}}\right)cos\left(\beta_{PD_{i}}\right),y_{PD}+sin\left(\alpha_{PD_{i}}\right)sen\left(\beta_{PD_{i}}\right),cos\left(\alpha_{PD_{i}}\right)\right).$$

Taking into account Equation (15), and that ∀i∈[1,4]: $\alpha_{PD_{i}}$ = $\alpha_{PR}$, in the expression (17), we obtain

(18) $$\begin{array}{c} (x_{PD_{i}},y_{PD_{i}},z_{PD_{i}})=\;\\ \left(x_{PD}+sin\left(\alpha_{PR}\right)cos\left(\frac{2\pi (i-1)}{4}\right),y_{PD}+sin\left(\alpha_{PR}\right)sen\left(\frac{2\pi (i-1)}{4}\right),cos\left(\alpha_{PR}\right)\right). \end{array}$$

Finally, $n_{PD}^{i}$ would be expressed as follows

(19) $$\begin{array}{c} n_{PD}^{i}=\;\\ \left(x_{PD}+sin\left(\alpha_{PR}\right)cos\left(\frac{2\pi (i-1)}{4}\right),y_{PD}+sin\left(\alpha_{PR}\right)sen\left(\frac{2\pi (i-1)}{4}\right),cos\left(\alpha_{PR}\right),\alpha_{PR},\frac{2\pi (i-1)}{4}\right). \end{array}$$

It is clear that the gain produced by angular diversity can be optimized if it is also given spatial diversity. However, this is outside the scope of this manuscript and could be an inconvenience in practice, since these $R_{x}$ will be located above the helmets of mining workers; therefore, all the properties of this ADR must be fulfilled with a compact structure design and minimal dimensions.

### 3.3. ADR with the Hemi-Dodecahedron Structure

To present a more efficient ICI mitigation alternative in underground mining VLC systems, a new ADR scheme that follows a regular hemi-dodecahedron structure is proposed in this work. This proposal fulfills all the general benefits of ADRs. Its geometric shape was chosen because it takes the best part of the ADRs of flat side faces, and of the ADRs of spherical surfaces, without overloading of PDs the $R_{x}$. With these features, we can generate greater granularity together with a compact design and easy insertion in a mining environment, specifically in the helmets of mining workers.

**Figure 6:**
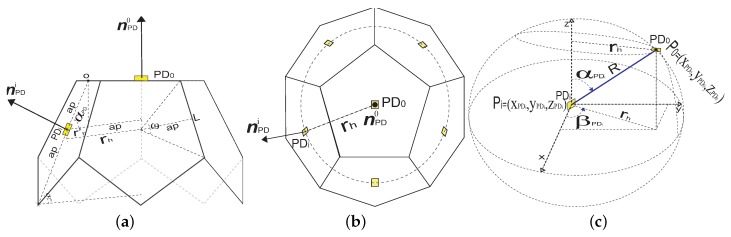
General structure of the hemi-dodecahedron ADR. (**a**) Side view, (**b**) top view, and (**c**) coordinate system of the PDs.

The physical structure of the proposed ADR is composed of six pentagonal faces, as we see in Figure 6b. Assuming that the ADR is always vertical, one of the faces will be pointing up, with a PD located in the center of its pentagonal surface, which we will call $PD_{0}$. The lateral faces, also of pentagonal surface, will be five in total and each one will have a PD on its surface, which we will call $PD_{i}$ with 1 ≤*i*≤ 5. As mentioned, all PDs, both those of the lateral faces and those of the upper face, will be located in the center of the pentagonal surfaces, all of them having the same FoV and optical and electrical performances. The location of $PD_{0}$ will be called $P_{0}$. The $PD_{i}$s will be located uniformly and equidistant from each other, in a circle whose center coincides in projection with $P_{0}$, and has radius $r_{h}$. The side and top view of the proposed ADR is shown in Figure 6a,b, respectively, with regular pentagonal surfaces of side *L*, central angle ω, and apothem ap.

By Geometrically, and since ω is the angle formed by two lines that join the pentagon center with two consecutive vertices, whose general expression is ω=2π/N, where *N* is the sides number of the polygon, it is possible to express ap in terms of ω and *L* as follows,

(20) $$ap=\frac{L}{2tan(\omega /2)}.$$

To maintain the same hybrid position and orientation coordinate system, and PDs normal vectors defined in Section 3.2, is necessary to define and delimit the elevation and azimuth angles that PDs can take. Thus, we will first define a normal vector $n_{PD}^{0}$ of $PD_{0}$ located at $P_{0}$ = $\left(x_{PD_{0}},y_{PD_{0}},z_{PD_{0}}\right)$, given as

(21) $$n_{PD}^{0}=\left(x_{PD_{0}},y_{PD_{0}},z_{PD_{0}},\alpha_{PD_{0}},\beta_{PD_{0}}\right),$$

and a normal vector $n_{PD}^{i}$ of $PD_{i}$ located at $P_{i}$ = $\left(x_{PD_{i}},y_{PD_{i}},z_{PD_{i}}\right)$, given as

(22) $$n_{PD}^{i}=\left(x_{PD_{i}},y_{PD_{i}},z_{PD_{i}},\alpha_{PD_{i}},\beta_{PD_{i}}\right).$$

Being $PD_{0}$, in the upper face, its inclination angle will be null, whereas the $PD_{i}$s in the lateral faces will have an inclination angle $\alpha_{PD_{i}}$∈[0,π]. We assume that due to the symmetry of regular polyhedra, all $PD_{i}$ will have the same $\alpha_{PD_{i}}$, which will be the same inclination angle, $\alpha_{p}$ as the pentagons of the lateral faces. Therefore, when the hemi-dodecahedron ADR is vertically oriented, $\alpha_{PD_{i}}$ will be defined as

(23) $$\alpha_{PD_{i}}=\left\{\begin{array}{cc} 0^{\circ }, & \mathrm{if}\;i=0\\ \alpha_{p}, & \mathrm{if}\;i=1,\cdots ,5 \end{array}\right..$$

According to the symmetric and equidistant distribution that each $PD_{i}$ will have in the circumference of radius $r_{p}$, we will define the azimuth angle as $\beta_{PD_{i}}$∈[0,2π]. As there are five lateral PDs, one on each side of the hemi-dodecahedron, we will determine that one of the PDs will have a fixed $\beta_{1}$, in $\beta_{PD_{1}}$=$0^{\circ }$. Due to this assumption, we can leave the general expression of $\beta_{PD_{i}}$ defined as

(24) $$\beta_{PD_{i}}=\frac{2\pi (i-1)}{5}\;\;\;\;\;\;\;\;for\;\;1\leq i\leq 5.$$

Thus, the PDs will be specifically oriented in 0, 2π/5, 4π/5, 6π/5, and 8π/5. It is possible to express $\beta_{PD_{i}}$ in terms of ω, taking this case, as the pentagon a polygon of *N* = 5.

(25) $$\beta_{PD_{i}}=\omega \left(i-1\right)\;\;\;\;\;\;\;\;for\;\;1\leq i\leq 5.$$

Thus, as $PD_{0}$ is located on the upper face of the hemi-dodecahedron, it will not have any rotation, so the azimuth angle $\beta_{PD_{0}}$, is not considered. Note that $\alpha_{PD_{i}}$ and $\beta_{PD_{i}}$ are important parameters in the physical design of the ADR. Therefore, and based on the ADR geometry, expressions that relate $PD_{0}$$PD_{i}$, $\alpha_{PD_{i}}$ and $\beta_{PD_{i}}$ angles can be derived. To present these expressions, we will use the Cartesian to spherical coordinate transformation tool, whose derivations and assumptions are presented below.

First, we will graphically establish the positions in the space of the PDs, with their respective angular and Cartesian coordinates, as we can see in Figure 6c; thus, the objective is to find an equivalence expressed as $\left(x_{PD},y_{PD},z_{PD}\right)$≡$\left(r_{h},\alpha_{PD},\beta_{PD}\right)$. We had previously defined the position of the $PD_{0}$, so we will now define the position of a $PD_{i}$ located on any of the ADR side faces, such as $P_{i}$=$\left(x_{PD_{i}},y_{PD_{i}},z_{PD_{i}}\right)$. In addition, we will define the Euclidean distance between $P_{0}$ and $P_{i}$ as *R* = $\left(x_{PD_{0}}-x_{PD_{i}},y_{PD_{0}}-y_{PD_{i}},z_{PD_{0}}-z_{PD_{i}}\right)$. According to the relation between Cartesian and spherical coordinates observed in Figure 6c, it is possible to express the coordinates of $P_{i}$ in terms of alpha and beta, as follows

(26) $$P_{i}=\left(x_{PD_{i}},y_{PD_{i}},z_{PD_{i}}\right)=\left(r_{h}cos\left(\beta_{PD_{i}}\right),r_{h}sen\left(\beta_{PD_{i}}\right),Rcos\left(\alpha_{PD_{i}}\right)\right).$$

Based on Figure 6a, a relation between $r_{h}$ and ap is obtained. We define $r_{h}^{\prime }$ as a segment of $r_{h}$ that is taken from $P_{i}$ to the downward projection of the junction between the side face where $P_{i}$ is located and the top face of the ADR. By trigonometric relationship, similarity of triangles and Thales theorem, we can obtain the following relation

(27) $$r_{h}^{\prime }=apcos\left(\alpha_{PD_{i}}\right),$$

(28) $$r_{h}=r_{h}^{\prime }+ap,$$

(29) $$r_{h}=ap\left[1+cos(\alpha_{PD_{i}})\right].$$

As expression (26) still has the component $z_{PD_{i}}$ in terms of *R*, it is necessary to find a relation between *R* and $r_{h}$, which is obtained by

(30) $$R=\frac{r_{h}}{sen(\alpha_{PD_{i}})}.$$

Thus, by replacing (29) and (30) in (26) we obtain

(31) $$P_{i}=\left(x_{PD_{i}},y_{PD_{i}},z_{PD_{i}}\right)=\left(ap\left[1+cos(\alpha_{PD_{i}})\right]cos\left(\beta_{PD_{i}}\right),ap\left[1+cos(\alpha_{PD_{i}})\right]sen\left(\beta_{PD_{i}}\right),\frac{ap\left[1+cos(\alpha_{PD_{i}})\right]}{sen(\alpha_{PD_{i}})}cos\left(\alpha_{PD_{i}}\right)\right).$$

Finally we add the coordinates’ reference of $P_{0}$, and replace Equation (20) in (31), to obtain $PD_{i}$ coordinates based on controllable design parameters, in the mathematical expression that follows,

(32) $$\begin{array}{c} P_{i}=\left(x_{PD_{i}},y_{PD_{i}},z_{PD_{i}}\right)=\\ (x_{PD_{0}}+\frac{L}{2tan(\omega /2)}\left[1+cos(\alpha_{PD_{i}})\right]cos\left(\beta_{PD_{i}}\right),y_{PD_{0}}+\frac{L}{2tan(\omega /2)}\left[1+cos(\alpha_{PD_{i}})\right]sen\left(\beta_{PD_{i}}\right),z_{PD_{0}}\\ -\frac{L}{2tan(\omega /2)}\left[1+cos(\alpha_{PD_{i}})\right]\frac{1}{tan(\alpha_{PD_{i}})}). \end{array}$$

According to this expression, and by finding the Cartesian and angular relationship, it is possible to optimize the size of the proposed ADR based solely on its $\alpha_{PD_{i}}$ and $\beta_{PD_{i}}$ angles; thus, trying to make it as compact and small as possible with *L*, considering that its application and location physics will be in a helmet of mining workers. However, this optimization is not the focus of this research, but it can be developed in future work. A factor to analyze is due to the existence of multiple PDs in the ADR; each of them will receive a light signal of LoS and NLoS paths with specific and different channel gains according to their location. Therefore, in conjunction with the ADRs analyzed in this section, mechanisms are needed to select the best of the received signals or combine them according to the parameters of the communication system. These mechanisms are called signal combining schemes, and we will present in the next section.

## 4. Signal Combining Schemes for ADR Solutions

The benefits and capabilities of ADRs to reduce ICI in underground mining VLC systems were presented in detail in Section 3.2 and Section 3.3. Therefore, as there are multiple PDs, it is necessary to obtain a single signal from the set of signals received by the ADR in $R_{x}$. In this section, we will explain mechanisms to obtain this signal, which is called signal combining schemes in $R_{x}$. To achieve this goal, and at the same time make a fair comparison between schemes; two scenarios will be presented, the first with a single PD in $R_{x}$, and the second with the analyzed ADRs equipped in $R_{x}$. In addition, being the overall coverage area of an ADR, the union of the coverage areas of each PD included in the ADR, it is necessary to adjust it to be the same area covered by an $R_{x}$ with a single PD. Therefore, each scenario presented in this section will have an $R_{x}$ with the same overall effective area, $N_{PD}$$A_{eff}$, which makes the comparison fair. Applying these criteria, we increase the active area of $R_{x}$ to improve the SINR with the geometry of the ADRs, increasing the reachable data rate while reducing the ICI.

By adjusting those physical parameters in both ADRs so that they can be comparable, the $R_{x}$ with more PDs can achieve a better SINR performance despite the fact that it receives a smaller portion of the desired signal. As the equivalent Θ of each ADR is also the same, when more PDs are on the same ADR, each PD will has a smaller Θ, which results in a smaller coverage area. Due to the diffusive nature of the reflected light, a smaller area means less reflected interference signal is picked up by each PD. Moreover, a larger number of PDs enables the system to benefit from better receiver diversity by means of the signal combining schemes, as long as we consider a limit on the number of PDs that we place. As a result, the pyramidal ADR and hemi-dodecahedron can be compared.

### 4.1. Receiver Scenario with a Single PD ($N_{PD}$ = 1)

In this scenario, the $R_{x}$ in the underground mining VLC environment will have a single PD installed. As one of the main objectives of this work is to mitigate the ICI in the mining VLC system (see Section 1), one of the most important communications metrics to verify the reduction of this problem is the SINR, which allows evaluation of the capacity and quality of the VLC channel. Thus, to maximize the SINR of the link that involves the $R_{x}$, the PD in it, must have the ability to choose from all the $T_{x}$s of the mining scenario, i.e., the one that achieved the best SINR. To accomplish this, we formulate an optimization problem, where the desired $T_{x}$, $T_{x_{a_{op}}}$, where $a_{op}$∈$\left[1,N_{T_{x}}\right]$, is described as [22,23,44]

(33) $$T_{x_{a_{op}}}=\underset{T_{x_{a_{op}}}}{arg\;max}\;\gamma_{{}_{T_{x_{a_{op}}},R_{x}}},$$

where $\gamma_{{}_{T_{x_{a_{op}}},R_{x}}}$ is the SINR of the link between $T_{x_{a_{op}}}$ and $R_{x}$. The SINR can be expressed as [22,23,44]

(34) $$\gamma_{{}_{T_{x_{a_{op}}},R_{x}}}=\frac{{\left(\tau \sigma \xi P_{a_{op}}H_{{}_{T_{x_{a_{op}}},R_{x}}}\right)}^{2}}{\sum_{a=1,a\neq a_{op}}^{N_{T_{x}}}{\left(\tau \sigma \xi P_{a}H_{{}_{T_{x_{a}},R_{x}}}\right)}^{2}+{\left(n_{R_{x}}\right)}^{2}},$$

where $H_{{}_{T_{x_{a_{op}}},R_{x}}}$ is the overall DC channel gain for underground mining VLC systems between $T_{x_{a_{op}}}$ and $R_{x}$, the sum term represents the interference produced by the $T_{x}$s that were not selected in the link, and $n_{R_{x}}$ is the total noise in $R_{x}$.

### 4.2. Receiver Scenario with Pyramidal ADR ($N_{PD}$ = 4) and Hemi-Dodecahedron ADR ($N_{PD}$ = 6)

In this scenario, the $R_{x}$ will be equipped with the ADRs described in Section 3.2 and Section 3.3. Therefore, we will have multiple received signals, one for each PD, to combine these signals into one, or select the best of them, three signal combining schemes, SBC, EGC, and MRC are described below.

#### 4.2.1. Select Best Combining Scheme

The SBC scheme is initially proposed in [44]. Adapted for our mining VLC system, the $R_{x}$, equipped with any of the ADRs presented, must select the best of the PDs in the ADR, which we will call $PD_{i_{op}}$ to establish a link with $T_{x_{a_{op}}}$. This process allows the fulfillment of the objective of maximizing the SINR, which is denoted in the optimization problem that follows [22,23,44]

(35) $$\left(T_{x_{a_{op}}},PD_{i_{op}}\right)=\underset{T_{x_{a_{op}}},PD_{i_{op}}}{arg\;max}\;\gamma_{{}_{T_{x_{a_{op}}},PD_{i_{op}}}},$$

where $\gamma_{{}_{T_{x_{a_{op}}},PD_{i_{op}}}}$ is the SINR of the link between $T_{x_{a_{op}}}$ and $PD_{i_{op}}$, denoted by

(36) $$\gamma_{{}_{T_{x_{a_{op}}},PD_{i_{op}}}}=\frac{{\left(\tau \sigma \xi P_{a_{op}}H_{{}_{T_{x_{a_{op}}},PD_{i_{op}}}}\right)}^{2}}{\sum_{a=1,a\neq a_{op}}^{N_{T_{x}}}{\left(\tau \sigma \xi P_{a}H_{{}_{T_{x_{a}},PD_{i_{op}}}}\right)}^{2}+{\left(n_{PD_{i_{op}}}\right)}^{2}}.$$

In this technique, ADRs will only consider the signal received by the PD that has the highest SINR. Therefore, for its physical implementation, it is necessary to install a circuit in the ADR that continuously monitors the SINR in each PD. In addition, a quick switching system is required for the selection of the desired PD. Theoretically, by selecting the best PD in the ADR, a higher SINR can be achieved; however, this scheme does not perform signal combination, thus the interference you will select will also be high.

#### 4.2.2. Equal Gain Combining Scheme

This scheme, proposed in [44], considers for our scenario the combination of the signals received in each PD of the ADRs in the simplest way, assigning a value (weight) to each of these signals. For the VLC system, this scheme must select the best $T_{x}$, $T_{x_{a_{op}}}$, under the same optimization problem described in (33). The basic principle of this technique is that the signals received by the PDs in the ADR installed in $R_{x}$, must be combined with the same weight; therefore, the SINR can be expressed as [22,23,44]

(37) $$\gamma_{{}_{T_{x_{a_{op}}},R_{x}}}=\frac{\tau \sigma \xi {\left(\sum_{i=1}^{N_{PD}}P_{a_{op}}H_{{}_{T_{x_{a_{op}}},PD_{i}}}\right)}^{2}}{\tau \sigma \xi \sum_{i=1}^{N_{PD}}\sum_{a=1,a\neq a_{op}}^{N_{T_{x}}}{\left(P_{a}H_{{}_{T_{x_{a}},PD_{i}}}\right)}^{2}+\sum_{i=1}^{N_{PD}}{\left(n_{PD_{i}}\right)}^{2}},$$

where $\sum_{i=1}^{N_{PD}}H_{{}_{T_{x_{a_{op}}},PD_{i}}}$ is the overall DC channel gain for underground mining VLC systems, which is obtained from the sum of the channel gains of all ADR PDs and $\sum_{i=1}^{N_{PD}}\sum_{a=1,a\neq a_{op}}^{N_{T_{x}}}{\left(P_{a}H_{{}_{T_{x_{a}},PD_{i}}}\right)}^{2}$ represents the total interference received by PDs. In terms of physical implementation of this scheme, an adder in the $R_{x}$ is necessary, which leads to a combiner circuit that must be installed in the ADRs. Although this scheme theoretically exceeds the SBC, when obtaining a better final signal, it is not effective to suppress the interference because each signal received in the PDs is valued with the same weight. Consequently, both the optimal signal and the interfering signals will be processed equally.

#### 4.2.3. Maximum Ratio Combining Scheme

This technique, proposed in [44], improves the EGC scheme due to the higher priority it gives to the PD signal that reaches the highest SINR. Therefore, like EGC, it assigns weights to the signals received by the ADR PDs, with the difference that the assigned weights will be proportional to the SINR that each signal obtains. For this scheme, like EGC, a $T_{x_{a_{op}}}$ is selected to maximize the SINR by following the expression (33). Therefore, the SINR is presented as follows [22,23,44],

(38) $$\gamma_{{}_{T_{x_{a_{op}}},R_{x}}}=\frac{\tau \sigma \xi {\left(\sum_{i=1}^{N_{PD}}P_{a_{op}}w_{{}_{T_{x_{a_{op}}},PD_{i}}}H_{{}_{T_{x_{a_{op}}},PD_{i}}}\right)}^{2}}{\tau \sigma \xi \sum_{i=1}^{N_{PD}}\sum_{a=1,a\neq a_{op}}^{N_{T_{x}}}{\left(P_{a}w_{{}_{T_{x_{a}},PD_{i}}}H_{{}_{T_{x_{a}},PD_{i}}}\right)}^{2}+\sum_{i=1}^{N_{PD}}\sum_{a=1,a\neq a_{op}}^{N_{T_{x}}}{\left(w_{{}_{T_{x_{a}},PD_{i}}}n_{PD_{i}}\right)}^{2}},$$

where *w* is the weight factor assigned to the signal received by the PDs, which can be calculated as [22,23,44]

(39) $$w_{{}_{T_{x_{a_{op}}},PD_{i}}}=\frac{{\left(\tau \sigma \xi P_{a_{op}}H_{{}_{T_{x_{a_{op}}},PD_{i}}}\right)}^{2}}{\sum_{a=1,a\neq a_{op}}^{N_{T_{x}}}{\left(\tau \sigma \xi P_{a}H_{{}_{T_{x_{a}},PD_{i}}}\right)}^{2}+{\left(n_{PD_{i}}\right)}^{2}}.$$

By assigning proportional weights to the SINR detected in each PD, this scheme enhances the optimal signal and attenuates the noise and interference components, thus reaching a high SINR. However, for the physical implementation in the studied ADRs, complex circuits are needed. In addition to monitoring the SINR in each PD, adding and multiplying the signals is required to achieve the combination proposed in this technique.

## 5. Results and Discussions

In this section, we focus on evaluating the performance of the illuminance conditions of the mining environments. In addition, we analyze the characteristics of the proposed VLC mining channel through the RMS Delay Spread. Finally, we evaluate the performance of the ADR solutions proposed in the system VLC, specifically in $R_{x}$ and applied in the two underground mining VLC scenarios, comparing them with a single PD, which is considered the baseline. This performance analysis is carry out and then discussed in terms of parameters, such as UDR, BER, and SINR based on an CDF. All evaluations are numerical and created with the use of simulations in Matlab software, in which the VLC scenarios were designed; comparisons are made between the proposed ADRs with their signal combining schemes and the single PD. The most relevant parameters used for the simulations development, with their respective values and references, can be seen in the Table 1.

### 5.1. Underground Mining Illuminance

**Figure 7:**
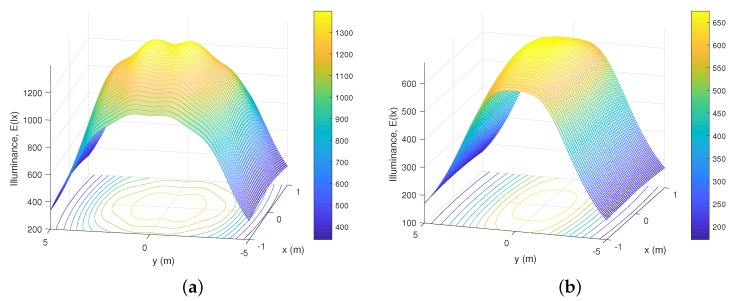
Illuminance distribution of underground mining VLC scenarios for the (**a**) mining roadway and (**b**) mine working face.

As we have presented in previous sections, one of the main benefits of VLC systems in underground mines is that they offer lighting and communication at the same time. Because in the system that we propose the optical $T_{x}$s are LED light sources, it is necessary to provide a mathematical lighting model for the $T_{x}$s. An important consideration is that this model is calculated only for direct light; therefore, in order to define the luminous flux *Q* as the optical power that the human eye can receive, the following expression is presented [11,16]

(40) $$Q=683N_{T_{x}}\int_{380}^{720}S\left(\lambda \right)V\left(\lambda \right)d\lambda ,$$

where V(λ) is the sensitivity function of the human eye and S(λ) is the distribution of the radiation spectrum. The relationship between *Q* and the illuminance I(ϕ) on the receiving surface is given by [11]

(41) $$I\left(\phi \right)=\frac{\delta Q}{\delta A_{p}}=\sum_{j=1}^{N_{T_{x}}}jI_{0}cos^{m}\left(\phi \right),$$

where $I_{0}$ is the center of light intensity of $T_{x}$. This expression allows us to calculate the light intensity in the reception plane of the $R_{x}$. However, a baseline is needed as a point of comparison for our analysis. Consequently, results are contrasted with the requirements of underground mining lighting standards, which indicate that the minimum light intensity requirements for these environments, is given by 0.158 W/m${}^{2}$ for the luminous power density, and 107.65 lux for the illuminance [46]. Considering these values, the illuminance is obtained by simulation both in the mining roadway scenario and in the mine working face, as we can see in Figure 7a,b, respectively. Further, the average height of the mining workers is also considered. As, the reception plane of the $R_{x}$ would be in their helmets, the illuminance is evaluated at 1.67 m above the floor in both scenarios. Also, all the parameters presented in Table 1 were considered for simulations.

Note in Figure 7a that the illuminance values are in the range of [400–1300] lux. It is also observed that the illuminance peaks occur precisely in the location of the $T_{x}$s, which are in a row in this scenario, taking into consideration that the $T_{x}$s are located at 3.7 m. Secondly, in Figure 7b, to obtain the illuminance values, the location of the $T_{x}$s changes to have a rectangular order, in addition to the height of the stage being increased to 4.7 m. Due to these variations, since the $T_{x}$s are further from the mining helmets, it is observed that the illuminance is in the range between [200 and 650] lux, which is less than the previous scenario. However, in both scenarios it is possible to note that the illuminance standard for underground mine environments is met.

### 5.2. RMS Delay Spread

The RMS delay spread is a parameter that allows us to quantify the temporal dispersion of a signal, in this case the optical signal, in indoor environments. For our study, this is specifically underground mining environments. Despite not being the focus of our work, it is important to make a brief analysis of the multi-path dispersion that exists in the scenarios presented due to the NLoS components. Therefore, the signals received in $R_{x}$, which are copies of the transmitted signals, they arrive at different times at $R_{x}$, due to the different lengths of the NLoS paths, which can induce ISI, significantly affecting the channel capacity and bandwidth. This is reflected in the maximum data rate ($R_{b}$) of the VLC channel, which will be limited by $R_{b}$≤ 1/(10 $D_{RMS}$) [11,49], where $D_{RMS}$ is the RMS delay spread. Therefore, time dispersion parameters are calculated with a truncation time ($T_{TR}$). The mean excess delay ($\mu_{RMS}$) and the $D_{RMS}$ can be represented in terms of the channel impulse response, and are given by the following expressions [11,49]

(42) $$\int_{0}^{T_{TR}}h^{2}\left(t\right)dt=0.97\int_{0}^{\infty }h^{2}\left(t\right)dt,$$

(43) $$\mu_{RMS}=\frac{\int_{0}^{\infty }t\;h^{2}\left(t\right)dt}{\int_{0}^{\infty }h^{2}\left(t\right)dt},$$

(44) $$D_{RMS}=\sqrt{\frac{\int_{0}^{\infty }{\left(t-\mu_{RMS}\right)}^{2}\;h^{2}\left(t\right)dt}{\int_{0}^{\infty }h^{2}\left(t\right)dt}}.$$

**Figure 8:**
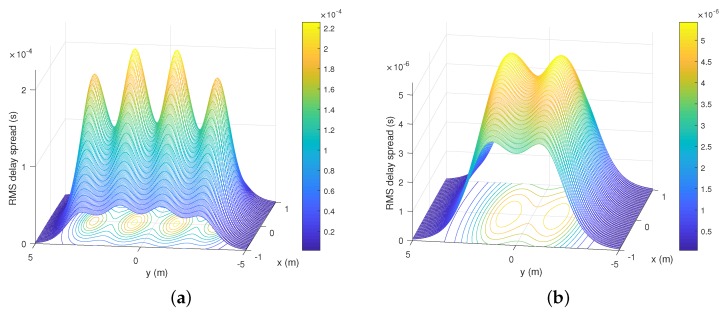
RMS delay spread distribution in the underground mining VLC scenarios. for the (**a**) mining roadway and (**b**) mine working face.

RMS delay spread for the mining roadway and mine working face scenarios are illustrated in Figure 8a,b, respectively. It can be noted that the maximum values are 2.208 × $10^{-4}$ s and 5.043 × $10^{-6}$ s for the first and second scenarios, respectively. Likewise, the minimum values are 0.213 × $10^{-4}$ s and 0.512 × $10^{-6}$ s, respectively. In addition, we observe in both scenarios that the RMS delay spread distribution is not uniform because the physical environments presented are not closed cuboids. Consequently, the LoS and NLoS components arrive at each scenario points at different times. However, it can be seen that the RMS delay spread decreases when the $R_{x}$ moves away from the $T_{x}$s.

### 5.3. User Data Rate (UDR)

Among the goals of our work (see Section 1) is the application of ADR solutions in $R_{x}$ to reduce ICI in underground mining environments. Therefore, it is necessary to use metrics to verify compliance with this objective. One of the characteristic parameters of performance measurements of communication systems, especially those of wireless communication, is the UDR, which allows us to analyze the maximum data transmission rate that can be achieved in the VLC scenarios. Thus, the mathematical expression that describes this metric is Shannon’s channel capacity as follows [43],

(45) $$R_{b}=log_{2}\left(1+\gamma \right).$$

Therefore, the ADR intends to maximize the γ of the mining VLC link, which is directly reflected in the decrease of the ICI, thus maximizing the channel capacity and improving the UDR. In this context, the γ of the pyramidal and hemi-dodecahedron ADRs, presented in Section 3.2 and Section 3.3, with each of the signal combining schemes, are analyzed and compared with this metric. The key parameters of the VLC system, the simulation scenarios developed for the two underground mining environments, and the parameters used in the ADRs are listed in Table 1.

To ensure that ADRs can effectively receive the signal of at least one $T_{x}$, and for a fair comparison, the ADRs half-angle FoV is fixed to $60^{\circ }$, being a typical value of the PDs we find in the market. The overall effective area ($N_{PD}$ $A_{p}$) for ADRs is also assumed to be of the same value. By varying the position of the $R_{x}$ around all possible locations in the underground mining scenarios, we can calculate the UDR.

**Figure 9:**
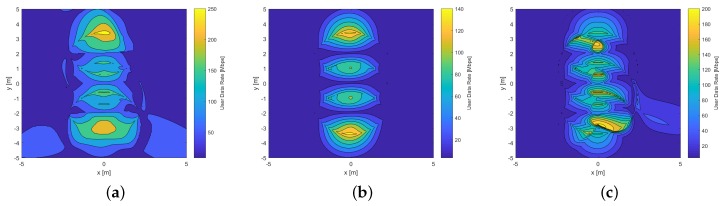
UDR distribution of the hemi-dodecahedron ADR in the mining roadway scenario: (**a**) MRC, (**b**) EGC, and (**c**) SBC schemes.

Figure 9 compares UDR distributions reached by the hemi-dodecahedron ADR in the mining roadway scenario. As we can see, all the signal combining schemes present a high data rate. However, to achieve these high data rates, it is necessary to reduce the ICI, which was achieved in this work. It is possible to infer, according to Figure 9, that when the $R_{x}$ is below a $T_{x}$s, it reaches the maximum UDR. This observation is valid for all the techniques analyzed. By comparing Figure 9a–c, the technique that achieves the highest data rate is MRC (a maximum value of 250 Mbps and a minimum value of 50 Mbps). In contrast, the technique that has the worst performance is the EGC (maximum value of 140 Mbps and minimum value of 20 Mbps). In addition, the lobes diameter of the MRC UDR distribution are the largest on the surface, which is also relevant as it will provide a higher transmission rate per area. This performance was expected because the mechanism used by this scheme also assigns weights to the interference, which improves the quality of the combined signal in the receiver.

**Figure 10:**
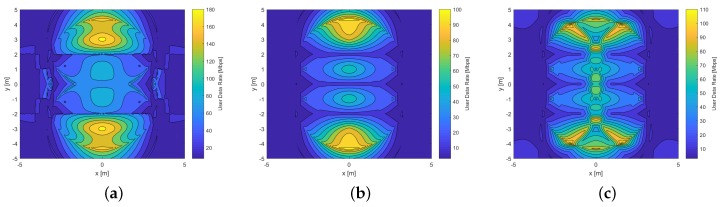
UDR distribution of the pyramidal ADR in the mining roadway scenario: (**a**) MRC scheme, (**b**) EGC scheme, and (**c**) SBC scheme.

Figure 10 compares UDR distributions reached by the pyramidal ADR in the mining roadway scenario. It is possible to recognize that there are similar characteristics with respect to Figure 9. However, if we compare the schemes of both Figure 9 and Figure 10, the hemi-dodecahedron ADR has larger lobes per area, in addition to reaching higher values of UDR, compared to the pyramidal ADR. This is due to the fact that the geometric distribution of the hemi-dodecahedron ADR has a better performance in the decrease of ICI and an increase of the SINR with respect to the pyramidal ADR. By comparing Figure 10a–c, the technique that achieves the highest data rate continues to be the MRC (a maximum value of 180 Mbps and a minimum value of 20 Mbps). In contrast, the technique that has the worst performance is the EGC (a maximum value of 100 Mbps and a minimum value of 10 Mbps).

**Figure 11:**
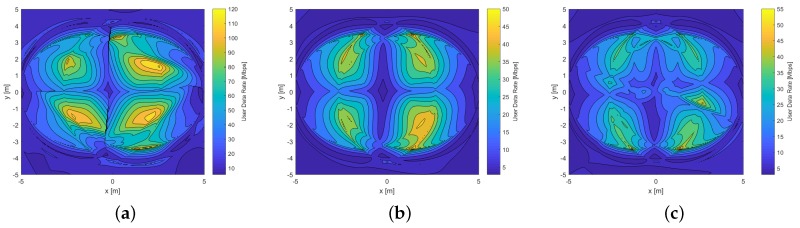
UDR distribution of the hemi-dodecahedron ADR in the mine working face scenario: (**a**) MRC, (**b**) EGC, and (**c**) SBC schemes.

Figure 11 compares UDR distributions reached by the hemi-dodecahedron ADR in the mine working face scenario. When changing the physical characteristics of this scenario, it is notorious that the UDR distributions for all signal combining schemes are different compared to the mining roadway scenario. The differences include the shape and location of the lobes and maximum UDRs reached in the environment, and mainly the drastic decrease in maximum UDRs for all techniques. This is due to the increase in distances between $T_{x}$s and the ADR in $R_{x}$, which causes the VLC channel to degrade and the SINR to decrease. By comparing Figure 11a–c, the technique that achieves the highest data rate continues to be the MRC (maximum value of 120 Mbps and minimum value of 20 Mbps). In contrast, the technique that has the worst performance is the EGC (maximum value of 50 Mbps and minimum value of 10 Mbps).

**Figure 12:**
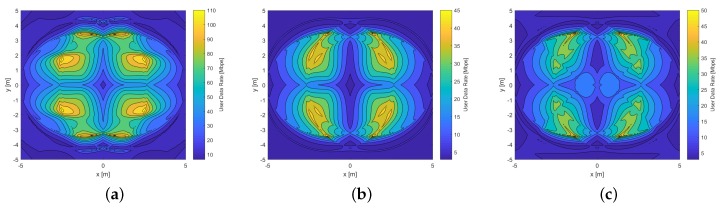
UDR distribution of the pyramidal ADR in the mine working face scenario: (**a**) MRC, (**b**) EGC, and (**c**) SBC schemes.

Figure 12 compares UDR distributions reached by the pyramidal ADR in the mine working face scenario. Comparing it with the hemi-dodecahedron ADR in this scenario, it presents similar characteristics in the UDR distribution; however, its performance is lower when reaching the maximum data rate. By comparing Figure 12a–c, the technique that achieves the highest data rate continues to be the MRC (a maximum value of 110 Mbps and a minimum value of 10 Mbps). In contrast, the technique that has the worst performance is the EGC (a maximum value of 45 Mbps and a minimum value of 5 Mbps).

### 5.4. Bit Error Rate

The modulation used in our work is the simplest modulation technique used in this type of VLC systems, which is called on-off keying (OOK), complying with the paradigm that VLC schemes must use IM in $T_{x}$ and DD in $R_{x}$. Additionally, and since it is not the focus of our research to analyze the modulation techniques that can be used, it is valid to use OOK to verify the BER parameter, which is expressed in terms of the distance between two bits. In the case of OOK modulation, as the $R_{x}$ observes which of the possible signals they handle is closer to the received signal, it is logical to think that there will be a lower error probability caused by noise and interference. Consequently, it is more efficient in terms of BER than other more complex modulations in indoor VLC environments.

**Figure 13:**
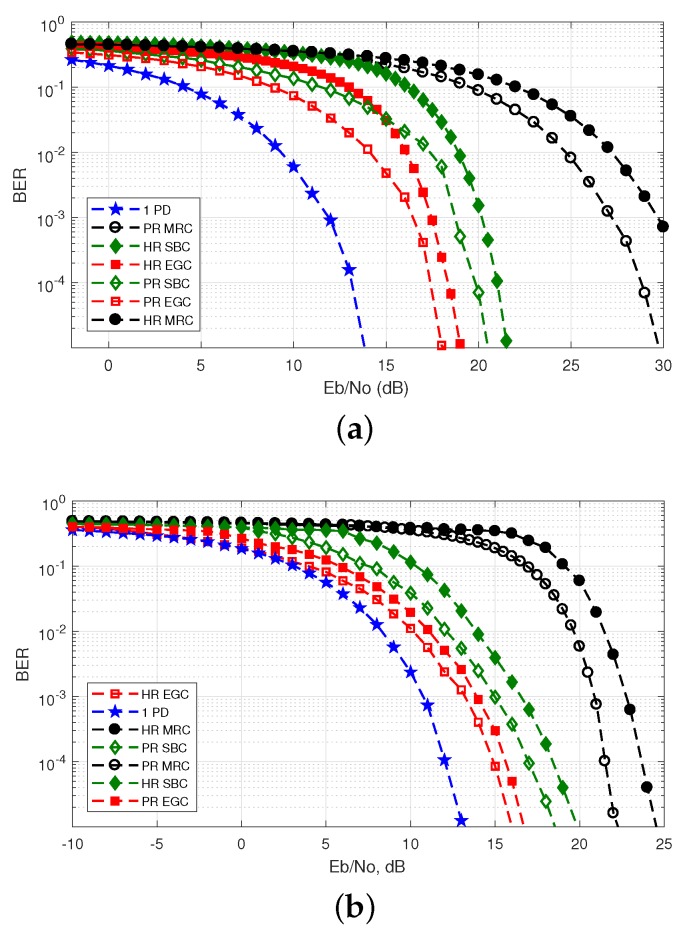
BER as a function of the relationship $E_{b}/N_{0}$ for the single PD, hemi-dodecahedron and pyramidal ADRs, with their signal combining schemes in the underground mining VLC scenarios. BER curves for the (**a**) mining roadway and (**b**) mine working face scenarios.

The BER was estimated via Monte Carlo simulations with the direct error counting method, i.e., 21 runs of $10^{5}$ bits were performed to have a confidence interval of 95% with an uncertainty factor of two on the error rate scale [11,50]. The simulated BER performance is shown in Figure 13 for a single PD, and the hemi-dodecahedron and pyramidal ADRs for different analyzed scenarios. This is for the corresponding signal combining scheme located in all possible positions of the mining roadway and mining working face.

We can see in Figure 13a the BER curves for the mining roadway scenario. The $R_{x}$ with a single PD has a poorer BER performance compared to the $R_{x}$s that have the hemi-dodecahedron and pyramidal ADRs. In contrast, the hemi-dodecahedron ADR with the MRC scheme has the best BER performance. For example, for a BER of $10^{-4}$, the $E_{b}/N_{0}$ improvement of this ADR are 3 dB and 19 dB, compared with the pyramidal ADR with the MRC scheme and the single PD, respectively. The results for the mine working face scenario are shown in Figure 13b. Similar to the previous scenario the hemi-dodecahedron ADR with the MRC scheme has the best BER performance. Specifically for a BER of $10^{-4}$, the $E_{b}/N_{0}$ improvement of this ADR are 2 dB and 11 dB, compared with the pyramidal ADR with the MRC scheme and the single PD, respectively. It can be seen that for both ADRs and for the only PD, the BER curves of this scenario have lower performance compared to the mining roadway scenario; this is due to the physical characteristics of $T_{x}s$ location and scenario dimensions, which causes the VLC channel to be degraded. Finally, the best BER performance in both scenarios is the ADR hemi-dodecahedron with the MRC scheme; this is due to its optimal physical construction and PD location characteristics, which allows for lower errors in data reception.

### 5.5. CDF of the SINR

**Figure 14:**
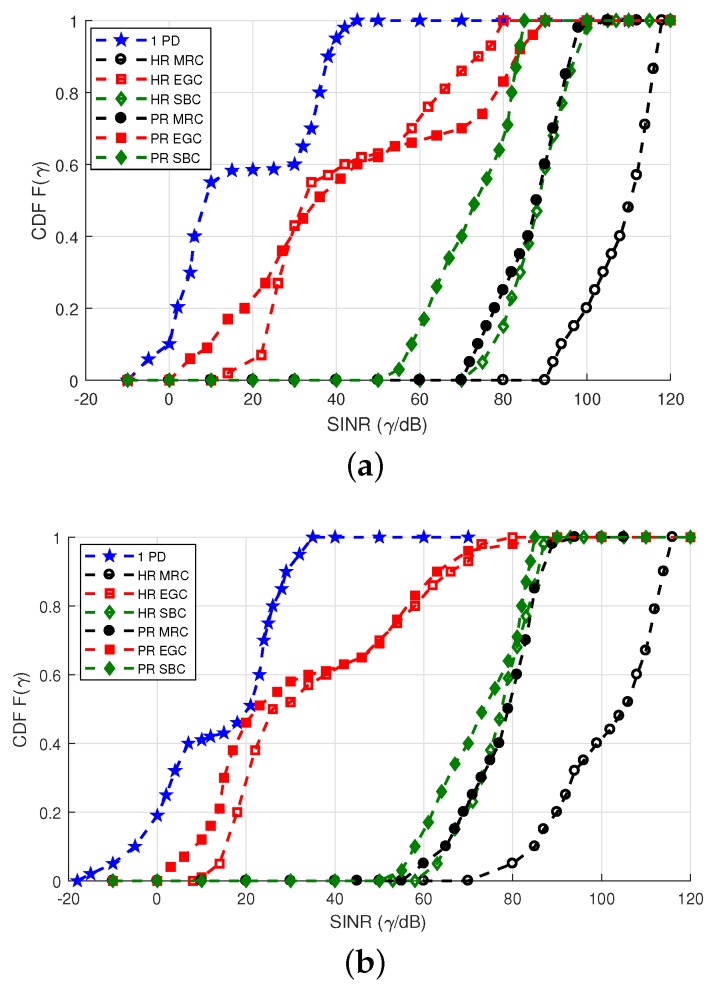
CDF of the SINR for the single PD, hemi-dodecahedron, and pyramidal ADRs, with their signal combining schemes, in the underground mining VLC scenarios. CDF of the SINR curves for the (**a**) mining roadway and (**b**) mine working face scenarios.

Another important parameter to determine the VLC system quality that we have adopted for underground mining scenarios is determined by the statistics of the received γ. Therefore, in this study, the CDFs of the γ received both in a single PD, and in the ADRs with their respective signal combining schemes, are empirically derived to evaluate the performance of the proposed solution. By varying the position of the $R_{x}$ through all possible positions in the two mining VLC scenarios presented, and estimating the respective attainable γ, it is possible to determine the CDF statistically, as illustrated in Figure 14.

Figure 14a compares the SINR performance of a single PD and the MRC, SBC, and EGC schemes for the hemi-dodecahedron and pyramidal ADRs, in the mining roadway scenario. The ADR that achieves better performance in the SINR with the three signal combination schemes is the ADR hemi-dodecahedron; this is due to its geometric distribution of PDs, although this angular diversity causes it to receive a smaller portion of the desired signal. Nevertheless, as the ADRs and the only PD have the same equivalent FoV, as there are more structurally located PDs, each of these PDs will have a smaller FoV and a smaller coverage area, which means that less interference signal is collected by PDs. For the considered LOS and NLoS channels, they can be divided into regions of high SINR and low SINR. $R_{x}$ in positions close to the center of the cell are free of ICI, which means that they can achieve a significantly higher SINR than users near the edge of the cell. The best performing scheme is the MRC in both ADRs, although the difference between the MRC in the hemi-dodecahedron ADR and the pyramidal ADR is approximately 20 dB; further, compared to a single PD, they have a difference of 80 dB and 50 dB, respectively. These results are since that the MRC scheme combines with a proportional weight the signals received in the different PDs, which increases the desired signal and attenuates the ICI. As a result, it achieves the best SINR performance. Figure 14b compares the SINR performance of a single PD and the MRC, SBC, and EGC schemes for the hemi-dodecahedron and pyramidal ADRs in the mine working face scenario. Compared to the mine roadway scenario, the performance of all the schemes in the two ADRs analyzed decreases. However, it maintains the trend that presents the hemi-dodecahedron ADR as the best performing Rx compared to the pyramidal ADR and the single PD, and the MRC as the scheme that has the largest SINR compared to the other schemes studied.

## 6. Conclusions

This manuscript presented an analysis of the ICI problem in underground mining VLC systems in two mining environments: mining roadway and mine working face. Further, a channel model that considers LoS and NLoS components was presented, as well as some propagation aspects. The main contribution of this manuscript is the proposal of two solutions in the receiver side to mitigate ICI. These solutions are based on hemi-dodecahedral and pyramidal ADRs. These techniques, in conjunction with three signal combination schemes, were applied in VLC underground mining environments via realistic simulation conditions. A first analysis of the scenarios included the illuminance and the RMS delay spread, which demonstrated that the minimum lighting standards in mines are met, and the RMS delay spread has a superior decrement when the distance between transmitter and the receiver increases, trend present in both scenarios. Finally, when comparing the pyramidal ADR and the hemi-dodecahedron ADR, the MRC scheme is the best option among the received signal combining schemes. On the other hand, regarding the use of ADRs, we show that the hemi-dodecahedron approach exceeds the pyramidal approach, in terms of user data rate with values between [250–50] Mbps and [120–20] Mbps for the mining roadway and mining working face scenarios, respectively. In terms of the BER, it presents $E_{b}$/$N_{0}$ values of 32 dB and 23 dB, when the BER gets a value of $10^{-4}$, for the mining roadway and mining working face, respectively. Whereas for the CDF of the SINR, it presents values between [120–90] dB and [118–75] dB for the mining scenarios roadway and mine working face, respectively.

To further validate the proposal, the development of experimental measurements in underground VLC mining environments is necessary. Additionally, ADR models could be optimized, in combination with other diversity schemes in the receiver side, to further mitigate ICI in these scenarios.

## Figures and Tables

**Table 1:** Underground mining VLC system simulation parameters.

System Model Parameters	Values	References
**Mining roadway**		
Dimensions (w×l×h)	(2×10×3.7) m	
Coordinates of the $T_{x}s\;\left(x,y,z\right)$	$T_{1}=\left(0,3,3.5\right),T_{2}=\left(0,1,3.5\right),T_{3}=\left(0,-1,3.5\right),$	
	$T_{4}=\left(0,-3,3.5\right)$	
**Mine working face**		
Dimensions (w×l×h)	(2×10×4.7) m	
Coordinates of the $T_{x}s\;\left(x,y,z\right)$	$T_{1}=\left(-0.5,-1.25,4.5\right),T_{2}=\left(0.5,1.25,4.5\right),$	
	$T_{3}=\left(0.5,-1.25,4.5\right),T_{4}=\left(-0.5,1.25,4.5\right)$	
**Other parameters**		
**Channel parameters**		
Absolute temperature	295 K	[45]
AWGN power spectral density	$2.5\times 10^{-23}A/\mathrm{Hz}$	[18]
Background dark current	10 nA	[45]
Boltzmann constant	$1.38\times 10^{-23}m^{2}\;\mathrm{kg}\;s^{-2}\;K^{-1}$	[45]
Capacitance	$112\times 10^{-8}\;F/m^{2}\;\left(s^{4}\;A^{2}\;m^{-4}\;{\mathrm{kg}}^{-1}\right)$	[45]
Electronic charge	$1.6\times 10^{-19}\;C$	[45]
FET channel noise factor	1.5	[45]
FET transconductance	0.03 S (kg${}^{-1}$ m${}^{-2}$ s${}^{3}$ A${}^{2}$)	[45]
Noise bandwidth	100 MHz	[45]
Noise bandwidth factor $I_{2}$	0.562	[45]
Noise bandwidth factor $I_{3}$	0.0868	[45]
**VLC transceiver parameters**		
Average output optical power	10 W	[19]
Band-pass filter of transmission	1	[9,16]
Gain of the optical filter	1	[23]
Helmet position	1.7 m
Modulation	OOK	[11]
Modulation bandwidth	50 MHz	[11]
Modulation index	0.3	[19]
Number of $R_{x}$s	1
Open-loop voltage gain	10	[45]
PD physical area	1 cm${}^{2}$	[9,11,16]
Optical filter bandwidth	340 nm to 694.3 nm	[48]
Optical filter center wavelength	340 ± 2 nm	[48]
Optical filter full width half max	10 ± 2 nm	[48]
Refractive index	1.5	[9,16]
Reflection coefficient	0.8	[9,11,16]
Responsivity	0.53 A/W	[18,19]
Rx FOV	$60^{\circ }$	[9,11,16]
Tx semi-angle at half power	$60^{\circ }$	[18,19]

## Abbreviations

The following abbreviations are used in this manuscript:

ADR	Angle diversity receiver
AWGN	Additive white gaussian noise
BER	Bit error rate
CDF	Cumulative distribution function
DC	Direct current
DD	Direct detection
EGC	Equal gain combining
FET	Field-effect transistor
FFR	Fractional frequency reuse
FoV	Field-of-view
ICI	Inter-cell interference
IM	Intensity modulation
ISI	Inter-symbol interference
I2M	Infrastructure-to-miner
JT	Joint transmission
LED	Light-emitting diode
LiFi	Light fidelity
LoS	Line-of-sight
MIMO	Multiple-input-multiple-output
MRC	Maximum ratio combining
M2M	Mining-to-miner
NLoS	Non-Line-of-sight
OOK	On-off keying
PD	Photo-diode
RF	Radio frequency
RMS	Root mean square
SBC	Select best combining
SER	Symbol error rate
SINR	Signal-to-interference-plus-noise-ratio
UDR	User data rate
VLC	Visible light communication
